# Time from presentation to pre-diagnostic chest X-ray in patients with symptomatic lung cancer: a cohort study using electronic patient records from English primary care

**DOI:** 10.3399/bjgp20X714077

**Published:** 2021-01-12

**Authors:** Kirsten D Arendse, Fiona M Walter, Mark Pilling, Yin Zhou, Willie Hamilton, Garth Funston

**Affiliations:** Primary Care Unit, Department of Public Health and Primary Care, University of Cambridge, Cambridge.; Primary Care Unit, Department of Public Health and Primary Care, University of Cambridge, Cambridge.; Primary Care Unit, Department of Public Health and Primary Care, University of Cambridge, Cambridge.; Wellcome Trust primary care doctoral fellow;; University of Exeter, Exeter.; Primary Care Unit, Department of Public Health and Primary Care, University of Cambridge, Cambridge.

**Keywords:** clinical practice guideline, diagnostic intervals, early diagnosis, lung cancer, chest X-ray

## Abstract

**Background:**

National guidelines in England recommend prompt chest X-ray (within 14 days) in patients presenting in general practice with unexplained symptoms of possible lung cancer, including persistent cough, shortness of breath, or weight loss.

**Aim:**

To examine time to chest X-ray in symptomatic patients in English general practice before lung cancer diagnosis, and explore demographical variation.

**Design and setting:**

Retrospective cohort study using routinely collected general practice, cancer registry, and imaging data from England.

**Method:**

Patients with lung cancer who presented symptomatically in general practice in the year pre-diagnosis and who had a pre-diagnostic chest X-ray were included. Time from presentation to chest X-ray (presentation–test interval) was determined and intervals classified based on national guideline recommendations as concordant (≤14 days) or non-concordant (>14 days). Variation in intervals was examined by age, sex, smoking status, and deprivation.

**Results:**

In a cohort of 2102 patients with lung cancer, the median presentation–test interval was 49 (interquartile range [IQR] 5–172) days. Of these, 727 (35%) patients had presentation–test intervals of ≤14 days (median 1 [IQR 0–6] day) and 1375 (65%) had presentation–test intervals of >14 days (median 128 [IQR 52–231] days). Intervals were longer among patients who smoke (equivalent to 63% longer than non-smokers; *P*<0.001), older patients (equivalent to 7% longer for every 10 years from age 27; *P* = 0.013), and females (equivalent to 12% longer than males; *P* = 0.016).

**Conclusion:**

In symptomatic primary care patients who underwent chest X-ray before lung cancer diagnosis, only 35% were tested within the timeframe recommended by national guidelines. Patients who smoke, older patients, and females experienced longer intervals. These findings could help guide initiatives aimed at improving timely lung cancer diagnosis.

## INTRODUCTION

In the UK, over 47 000 patients are diagnosed with lung cancer each year and the disease is the leading cause of cancer mortality, accounting for 21% of all UK cancer-related deaths.^1^^,^^2^ Though lung cancer survival rates in the UK have improved over the last decade, they remain less favourable than in other Northern and Western European countries, partly due to the more advanced stage at diagnosis in UK patients.^3^^,^^4^ Missed diagnostic opportunities, which can result from interactions between patient and healthcare practitioner, and health-system factors, may contribute to late-stage diagnosis and poor cancer outcomes.^5^^,^^6^

Achieving more timely cancer diagnosis is a key strategy of the NHS.^7^ This strategy is supported by the National Institute for Health and Care Excellence (NICE) cancer guidelines, which provide evidence-based recommendations to GPs in England, Wales, and Northern Ireland on the investigation and referral of patients with symptoms of possible cancer.^8^^,^^9^ However, few studies have assessed how frequently these guidelines are followed. With limited evidence on guideline concordance, assessing the impact of recommendations is challenging.

Previous studies have investigated timeliness of diagnostic activities for lung cancer that occur after referral from primary care, such as waiting times for specialist appointments.^10^^–^^13^ However, little is known about potential missed diagnostic opportunities in primary care when patients first present with symptoms. An improved understanding of how patients with cancer-associated features are managed in primary care before referral to specialist care could help identify missed diagnostic opportunities and guide interventions aimed at improving the timeliness of cancer diagnosis and treatment.^5^

Chest X-ray was recommended as the first-line investigation in patients presenting with features of possible lung cancer in the 2005 NICE guidelines and the revised 2015 guidelines.^8^^,^^9^ They recommended that chest X-ray is performed within 14 days of symptomatic presentation, including persistent cough, shortness of breath, or weight loss.^8^^,^^9^ This study aimed to examine the time to chest X-ray in symptomatic patients presenting in English general practice before lung cancer diagnosis, and to determine what proportion of patients had a chest X-ray within the recommended 14-day timeframe. In addition, the authors sought to explore how time to chest X-ray varied with age, sex, smoking status, and deprivation level.

## METHOD

### Study design and population

This retrospective cohort study utilised routinely collected datasets from NHS patients in England. This included primary care data from the Clinical Practice Research Datalink (CPRD), cancer registry data from the National Cancer Registration and Analysis Service (NCRAS), and imaging data from the Hospital Episode Statistics Diagnostic Imaging Dataset (HES DID). The CPRD consists of anonymised, coded data collected from GP records, including information on demographics, symptoms, and diagnoses. The CPRD contains data on some 11 million patients and is broadly representative of the UK population.^14^ NCRAS data contain information on patients diagnosed with cancer including diagnosis date, tumour type, and stage. HES DID data contain imaging information, including test type and imaging dates for patients undergoing imaging in NHS hospitals.^15^^,^^16^ Datasets for this study were linked at patient level by NHS digital.^16^

**Table:** How this fits in

England’s national cancer referral guidelines recommend that patients attending general practice with unexplained symptoms possibly caused by lung cancer, such as persistent cough, shortness of breath, and weight loss, have a chest X-ray promptly (within 14 days) to aid timely diagnosis. Only 35% of patients with lung cancer in this study had a chest X-ray within the recommended 14 days; and time between attending general practice with symptoms and having an X-ray was longer among people who smoke, females, and older patients. This research highlights a potential source of delayed lung cancer diagnosis and could inform initiatives aiming to achieve earlier diagnosis and improve outcomes.

### Patient sample

This research forms part of a broader study (protocol number: 17_107R). The cohort for the broader study includes patients with a code for any of the 11 common cancers recorded in CPRD between 1 April 2012 and 31 December 2015. From this baseline cohort, patients were included if they had a code for lung cancer in NCRAS. Date of diagnosis was taken as the first record of lung cancer in NCRAS rather than CPRD because NCRAS uses a hierarchical approach for determining diagnosis dates.^17^

Patients were included if they:

- had a new record of primary lung cancer;
- presented with symptoms and/or signs of possible lung cancer in the year before; diagnosis (Box 1); and
- had a pre-diagnostic chest X-ray after first symptomatic presentation.

**Box 1.** National Institute for Health and Care Excellence guidelines for referral of suspected cancer (2005)^8^

**Section 1.3: Lung cancer**
**Specific recommendations** 1.3.2. An urgent referral for a chest X-ray should be made when a patient presents with: haemoptysis, or any of the following unexplained persistent, that is, lasting >3 weeks, symptoms and signs: chest and/or shoulder pain, dyspnoea, weight loss, chest signs, hoarseness, finger clubbing, cervical and/or supraclavicular lymphadenopathy, cough with or without any of the above, features suggestive of metastasis from a lung cancer, for example, in brain, bone, liver, or skin. 1.3.3. An urgent referral should be made for either of the following: persistent haemoptysis in smokers or ex-smokers who are aged ≥40 years; or a chest X-ray suggestive of lung cancer (including pleural effusion and slowly resolving consolidation). 1.3.4. Immediate referral should be considered for the following: signs of superior vena caval obstruction (swelling of the face and/or neck with fixed elevation of jugular venous pressure); or stridor.
**Referral timelines** The referral timelines used in this guideline are as follows: **Immediate:** an acute admission or referral occurring within a few hours, or even more quickly if necessary. **Urgent:** the patient is seen within the national target for urgent referrals (currently 2 weeks).

### First presentation

Symptomatic presentations were identified from CPRD data using a predeveloped list of codes for the lung cancer symptoms and signs from the 2005 NICE guidelines.^18^^–^^20^ This includes haemoptysis, dyspnoea, weight loss, hoarseness, and cough (Box 1).^8^ As in similar studies, first presentation was taken as the first recorded symptom or sign of possible lung cancer in the year pre-diagnosis.^18^^,^^19^ Patients without a recorded symptomatic presentation in the year pre-diagnosis were excluded. The authors also excluded people who smoke or ex-smokers aged ≥40 years with haemoptysis, and patients with superior vena caval obstruction and stridor, as NICE guidelines recommend direct specialist referral rather than chest X-ray for these patients (Box 1).^8^ The number of recorded symptoms and signs on the date of first presentation was documented as 1 or >1 for analysis.

### Chest X-ray

**Figure 1.**
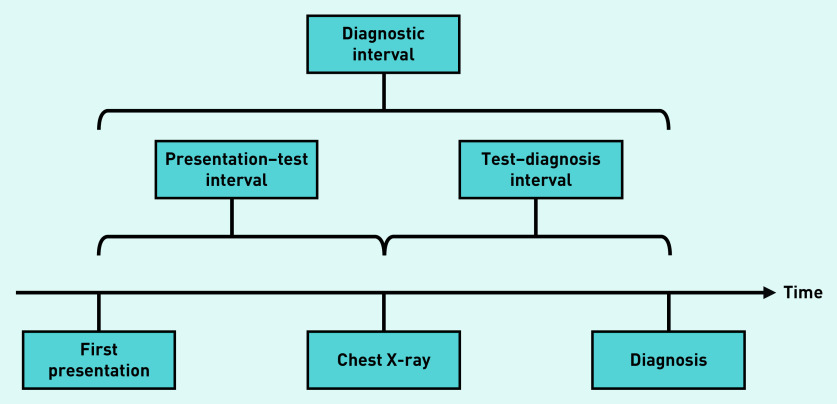
**Evaluated time intervals from presentation to diagnosis.**

Patients with a pre-diagnostic chest X-ray recorded in HES DID after first presentation were included. Time from first symptomatic presentation to chest X-ray, the ‘presentation–test’ interval was calculated for each patient (Figure 1). Presentation–test intervals of ≤14 days were considered guideline concordant, as NICE 2005 guidelines recommended a chest X-ray within 14 days of presentation.^8^

### Demographics

Patients were categorised as ‘smoker’, ‘ex-smoker’ or ‘non-smoker’ using their most recent smoking code before chest X-ray (see Supplementary Appendix S1). As NICE guidelines made recommendations for investigation and referral based on smoking status, patients without smoking data were excluded.^8^ Only the year of birth for each patient was available. The authors assigned all patients the birth date of 1 July. The index of multiple deprivation was documented in quintiles, 1 being the least and 5 the most deprived.

### Statistical analysis

The association between presentation–test interval duration (number of days as an integer), and sex, age, smoking, and deprivation was investigated. Age and sex were evaluated because of their association with timeliness in cancer diagnosis in previous studies.^10^^,^^19^ Deprivation was included because lung cancer mortality rates are highest among deprived groups.^21^^,^^22^ Smoking was included because of its strong link to respiratory comorbidity and its importance in lung cancer aetiology.^23^^,^^24^ Number of clinical features were accounted for at first presentation as multiple features are more predictive of lung cancer than single features.^25^

Unadjusted analyses were performed to explore the isolated effect of each variable and adjusted multivariable regression to explore combined effects. Regression model diagnostics were examined and assumptions were satisfied, that is, negative binomial dispersion parameter alpha was significant and residual plot was satisfactory. Incidence rate ratios (IRR) were calculated to determine relative effects of variables on presentation–test interval duration using negative binomial regression. Variables with an IRR >1 were interpreted as being associated with more days between presentation and chest X-ray, which is equivalent to longer intervals. For example, an IRR of 1.5 = 50% more days, which is equivalent to a 50% longer interval. A Cox proportional-hazards model was fitted as a sensitivity analysis and gave very similar conclusions.

All data management and analyses were conducted using Stata (version 15.1).

## RESULTS

### Patient cohort and demographics

**Figure 2.**
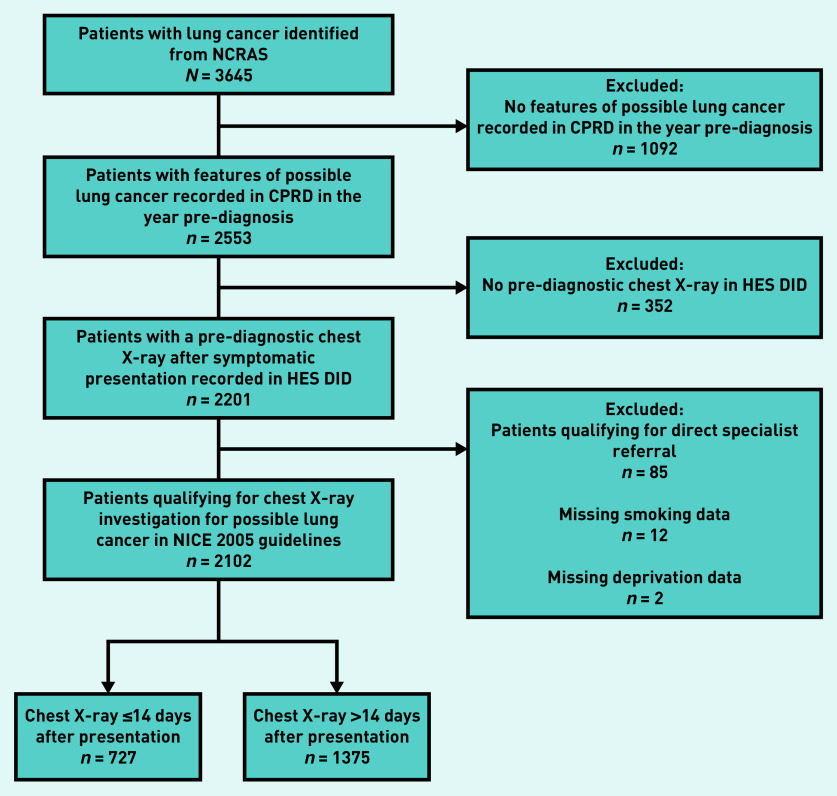
**Application of study inclusion criteria. CPRD = Clinical Practice Research Datalink. HES DID = Hospital Episode Statistics Diagnostic Imaging Dataset. NCRAS = National Cancer Registration and Analysis Service. NICE = National Institute for Health and Care Excellence.**

A total of 3645 patients had a new lung cancer diagnosis during the study period, of whom 2553 (70%) presented with features of possible lung cancer in the year prediagnosis. Of these, 2201 patients (86%) had a pre-diagnostic chest X-ray after presentation. A further 99 patients were excluded: 85 (4%) qualified for direct specialist referral, two had missing deprivation data, and 12 had missing smoking data (<1%) (Figure 2). The final cohort (*n* = 2102) had a median age of 72 years. Of these, 1148 patients (55%) were male. The majority were ex-smokers (56%, *n* = 1168) or smokers (37%, *n* = 785). Patient demographics are summarised in Table 1.

**Table 1.** Proportion of NICE-guideline concordant pre-diagnostic chest X-rays (≤14 days after presentation) among symptomatic patients with lung cancer by sociodemographic factors

	**Patients in cohort, *n* (%)**		**Total, *N***

	**Chest X-ray ≤14 days (guideline concordant)**	**Chest X-ray >14 days (guideline non-concordant)**	
**All**	727 (35)	1375 (65)	2102

**Sex**			
Male	426 (37)	722 (63)	1148
Female	301 (32)	653 (68)	954

**Age categories, years**			
<40	1 (33)	2 (67)	3
40–49	25 (48)	27 (52)	52
50–59	85 (35)	156 (65)	241
60–69	214 (37)	362 (63)	576
70–79	240 (32)	517 (68)	757
80–89	142 (34)	279 (66)	421
≥90	20 (38)	32 (62)	52

**Deprivation quintile**			
1 (least deprived)	133 (36)	234 (64)	367
2	147 (40)	223 (60)	370
3	141 (33)	280 (67)	421
4	152 (35)	286 (65)	438
5 (most deprived)	154 (30)	352 (70)	506

**Smoking status**			
Non-smoker	71 (48)	78 (52)	149
Ex-smoker	396 (34)	772 (66)	1168
Smoker	260 (33)	525 (67)	785

**Number of features on first presentation**			
1	692 (35)	1285 (65)	1977
>1	35 (28)	90 (72)	125

### Stage and histology

Most patients had a histological (*n* = 1479, 70%) or cytological (*n* = 217, 10%) diagnosis. In total, 1472 (70%) had non-small-cell lung cancer, 249 (12%) had small-cell lung cancer and 381 (18%) were unspecified. Stage was documented for 1959 (93%) patients; most had stage III (*n* = 490; 25%) or stage IV (*n* = 1019; 52%) lung cancer (data not shown).

### Time to chest X-ray and guideline concordance

The overall median presentation–test interval was 49 (interquartile range [IQR] 5–172) days. A total of 727 (35%) patients had a presentation–test interval ≤14 days, that is, guideline concordant (median 1 [IQR 0–6] day). The remaining 1375 (65%) patients had a presentation–test interval of >14 days (median 128 [IQR 52–231] days), that is, guideline non-concordant (Table 1).

### Demographic variation in intervals

Median presentation–test intervals are displayed by demographics in Table 2. Multivariable regression showed longer presentation–test intervals among smokers than non-smokers (IRR 1.63, 95% confidence interval [CI] = 1.29 to 2.05; equivalent to 63% longer; *P*<0.001), older patients (IRR 1.07, 95% CI = 1.01 to 1.12, for every additional 10 years from age 27; equivalent to 7% longer; *P* = 0.013) and females (IRR 1.12, 95% CI = 1.02 to 1.24; equivalent to 12% longer than males; *P* = 0.016). Deprivation was weakly associated with presentation–test intervals, showing longer intervals with greater deprivation. Presentation–test intervals were longer among patients with >1 symptom on presentation, though this association was weak (IRR 1.20, 95% CI = 1.00 to 1.43; equivalent to 20% longer; *P* = 0.052).

**Table 2.** Association between demographics and presentation–test intervals using negative binomial regression^a^

	**Patients in cohort**		**Presentation–test interval, days**	
	
	***n* (%)**	**Median (IQR)**	**IRR (adjusted)^b^**	**95% CI (*P*-value)**
**Total**	2102 (100)	49 (5–172)	–	–

**Constant**	–	–	35.77	23.09 to 55.40 (<0.001)

**Sex**				
Male	1148 (55)	41 (4–162)	Ref	–
Female	954 (45)	63 (7–182)	1.12	1.02 to 1.24 (0.016)

**Age**				
For every additional10 years from age 27	–	–	1.07	1.01 to 1.12 (0.013)

**Deprivation quintile**				
1 (least deprived)	367 (17)	43 (4–162)	Ref	–
2	370 (18)	37 (3–147)	0.86	0.73 to 1.02 (0.081)
3	421 (20)	46 (6–173)	1.01	0.86 to 1.18 (0.927)
4	438 (21)	49 (6–184)	1.03	0.88 to 1.21 (0.704)
5 (most deprived)	506 (24)	73 (9–184)	1.08	0.93 to 1.25 (0.326)

**Smoking status**				
Non-smoker	149 (7)	18 (1–85)	Ref	Ref
Ex-smoker	1168 (56)	52 (6–173)	1.58	1.26 to 1.99 (<0.001)
Smoker	785 (37)	55 (6–181)	1.63	1.29 to 2.05 (<0.001)

**Number of features on first presentation**				
1	1977 (94)	48 (5–170)	Ref	Ref
>1	125 (6)	91 (10–190)	1.20	1.00 to 1.43 (0.052)

**ln (alpha)**	–	–	0.82	0.77 to 0.88

a Likelihood ratio (LR) test of alpha = 0, χ^2^ = 240 000, P<0.001.

b Variables included in the adjusted analysis include sex, age in years, level of deprivation, smoking status, and number of features at first presentation.

IQR = interquartile range. IRR = incidence rate ratio. ln (alpha) = natural log of alpha. Alpha is the over-dispersion parameter (values >0 indicate the distribution’s variance is greater than its mean).

## DISCUSSION

### Summary

In this study, only 35% of patients with lung cancer had a pre-diagnostic chest X-ray within the NICE-recommended 14-day timeframe following symptomatic presentation to primary care. Time between symptomatic presentation and chest X-ray was longer among females, smokers, and older patients, reflecting a lower rate of guideline concordance in these groups. These findings highlight a potential missed opportunity for timely lung cancer diagnosis in England and could be used to guide interventions aimed at improving outcomes, particularly targeting sociodemographic disparities in healthcare access and quality.

### Strengths and limitations

This study’s strengths include the large sample size, high proportion of complete data, the retrospective cohort design, and originality. The reliance on codes to identify presentations in CPRD is a limitation because information about duration was unavailable. The 2005 NICE guidelines recommend chest X-ray if patients had ‘persistent’ features, that is, ≥3 weeks.^8^ The authors assumed that symptoms and signs recorded within CPRD had been present for this time period, however, some patients may have been symptomatic for <3 weeks, which could contribute to an underestimation of guideline concordance.

Some patients had no pre-diagnostic chest X-ray in HES DID; the authors were unable to identify whether this resulted from no prediagnostic X-ray or no recorded data. The cohort was restricted to patients diagnosed with lung cancer; patients without lung cancer who had symptomatic presentations warranting a chest X-ray were not evaluated. Therefore, the researchers were unable to assess overall concordance with guideline-recommended X-ray timeframes, only concordance in those subsequently diagnosed with lung cancer. Nevertheless, the group evaluated is the most likely to benefit from timely investigation and diagnosis.

The effect of comorbid diseases on presentation–test intervals was not investigated, but the effect of smoking was investigated, which is strongly related to key comorbidities including chronic obstructive pulmonary disease (COPD). Previous studies found that comorbidities are associated with longer secondary care intervals in lung cancer and longer diagnostic intervals in other cancer types.^13^^,^^26^^,^^27^ Further research investigating the relationship between comorbidities and presentation–test intervals in lung cancer is needed.

### Comparison with existing literature

A previous study reported no significant change in lung cancer diagnostic intervals after the introduction of the 2005 NICE guidelines, which may in part be explained by the low concordance with guideline recommendations on chest X-ray investigations observed in the present study.^18^ Nicholson *et al* found that GPs in the UK only followed national guidelines when investigating possible lung cancer 47% of the time, lower than non-UK jurisdictions (58%).^28^ In contrast, Baughan *et al* found a high rate (90.9%) of compliance with national referral guidelines for any cancer.^12^ The validity of these studies is limited by recall bias and missing information as both analysed self-evaluated accounts of compliance.

The low guideline concordance identified in the present study is likely to be due to interacting patient, clinician, and health system factors. Health systems can contribute to delays if demand for services exceeds available resources, for example, lack of imaging equipment and professionals to perform tests.^5^^,^^29^ Difficulty accessing X-rays from primary care could also extend intervals. In one study, 32% of GPs reported waiting ≥1 week to obtain a chest X-ray.^30^ While such system delays may lengthen presentation–test intervals, they are likely to affect all groups and therefore do not necessarily explain differences in interval length between demographic subgroups, for example, males and females. Lung cancer poses a real diagnostic challenge in general practice as patients usually present with ‘low-risk but not no-risk’ clinical features, most frequently cough or dyspnoea, which are common in non-malignant conditions.^31^ This is demonstrated in the present study as only 85 of 2201 (4%) patients presented with high-risk presentations warranting specialist referral and were therefore excluded. Diagnostic difficulty can lead to multiple pre-diagnostic consultations and potential diagnostic delay, particularly among individuals with comorbidities.^25^^,^^32^ Comorbid disease, most evidently COPD, may also mask symptoms of lung cancer and lengthen intervals.^6^^,^^33^^–^^35^

Associations between timely lung cancer diagnosis in the UK and sociodemographic factors have been evaluated in previous studies.^10^^,^^19^^,^^36^ As in the present study, age inequalities in cancer services have been previously reported, with evidence of longer intervals and lower proportions of urgent referrals among older patients.^10^^,^^12^^,^^19^^,^^37^ Authors have suggested that this may reflect a tendency towards therapeutic nihilism or discrimination against older patients, whose care is often determined by age rather than need, despite cancer being more common in this population.^12^^,^^19^^,^^36^ Furthermore, age-related comorbidities may contribute to delays and accessibility issues.^19^^,^^38^ Din *et al* noted longer lung cancer diagnostic intervals among females, as observed in the present study.^19^ GP behaviour may be influenced by sex, with symptoms treated as more serious in males prompting more rapid investigation.^5^^,^^19^ GPs may consider the higher incidence of lung cancer in males (particularly historically) when considering whether to request investigations.^1^ The longer time to chest X-ray experienced by smokers could result from their increased risk of respiratory comorbidity (a potential confounding factor).^23^ In addition, stigmatisation of smoking may influence patients’ decisions to return for chest X-ray after being informed of suspected lung cancer.^39^ The present finding that greater deprivation is only weakly associated with longer test–diagnostic intervals is in keeping with a recent systematic review conducted by Forrest *et al*.^36^ Though other evidence shows that higher deprivation is associated with increased lung cancer incidence and mortality, its relationship to risk and outcomes is complex.^21^^,^^22^ It is thought that social class contributes to the differences in smoking that drives health inequalities between the least and most deprived groups, particularly impacting the prevalence of lung cancer.^40^ Accounting for smoking, a potential mediating factor, could partly explain why a weak association between deprivation and interval duration was found in the present study.

### Implications for research and practice

It is thought that concordance with NICE and other evidence-based guidelines will improve timely cancer diagnosis, though the supporting evidence for lung cancer is lacking.^7^^,^^18^^,^^41^^–^^44^ Longer time intervals allow tumour growth and disease progression and thus could contribute to poorer lung cancer survival.^2^^,^^45^^–^^47^ This suggests that the two-thirds (65%) of patients with lung cancer who did not receive guideline concordant care in the present study may have experienced a missed diagnostic opportunity, and may experience poorer outcomes, including reduced survival, as a result. However, further research exploring the effect of longer presentation–test intervals on stage and survival would be needed to confirm this. As discussed, the poor overall concordance with recommended intervals for chest X-ray are unlikely to be due to a single cause but rather a range of factors. Given the importance of early lung cancer diagnosis, further research is needed to identify causes for low guideline concordance and to address them. The present findings support theories that older patients, females, and smokers are more likely to experience delayed diagnosis and missed diagnostic opportunities.^19^^,^^25^^,^^32^^,^^35^ These findings could inform future guidelines, education programmes, and early diagnostic initiatives. Further research, aimed at understanding the mechanisms by which presentation–test intervals are prolonged in the patient groups identified in this study, could help direct strategies aimed at reducing diagnostic delay in lung cancer.
